# Genetics, environmental stress, and amino acid supplementation affect lactational performance via mTOR signaling pathway in bovine mammary epithelial cells

**DOI:** 10.3389/fgene.2023.1195774

**Published:** 2023-08-10

**Authors:** Bin Li, Muhammad Zahoor Khan, Ibrar Muhammad Khan, Qudrat Ullah, Zhuo-Ma Cisang, Nan Zhang, Dan Wu, Bingjian Huang, Yulin Ma, Adnan Khan, Nan Jiang, Muhammad Zahoor

**Affiliations:** ^1^ Institute of Animal Husbandry and Veterinary, Tibet Autonomous Regional Academy of Agricultural Sciences, Lhasa, China; ^2^ Liaocheng Research Institute of Donkey High‐Efficiency Breeding and Ecological Feeding, Agricultural Science and Engineering School, Liaocheng University, Liaocheng, China; ^3^ Faculty of Veterinary and Animal Sciences, The University of Agriculture, Dera Ismail Khan, Pakistan; ^4^ Anhui Province Key Laboratory of Embryo Development and Reproduction Regulation, Anhui Province Key Laboratory of Environmental Hormone and Reproduction, School of Biological and Food Engineering, Fuyang Normal University, Fuyang, China; ^5^ Tibet Autonomous Region Animal Husbandry Station, Lhasa, China; ^6^ College of Life Sciences, Liaocheng University, Liaocheng, China; ^7^ State Key Laboratory of Animal Nutrition, Beijing Engineering Technology Research Center of Raw Milk Quality and Safety Control, College of Animal Science and Technology, China Agricultural University, Beijing, China; ^8^ Genome Analysis Laboratory of the Ministry of Agriculture, Agricultural Genomics Institute at Shenzhen, Chinese Academy of Agricultural Sciences, Shenzhen, China; ^9^ Department of Molecular Medicine, Institute of Basic Medical Sciences, University of Oslo, Oslo, Norway

**Keywords:** cow mammary epithelial cells, mTORC1 signaling pathway, amino acids, environmental stress, milk production

## Abstract

Mammary glands are known for their ability to convert nutrients present in the blood into milk contents. In cows, milk synthesis and the proliferation of cow mammary epithelial cells (CMECs) are regulated by various factors, including nutrients such as amino acids and glucose, hormones, and environmental stress. Amino acids, in particular, play a crucial role in regulating cell proliferation and casein synthesis in mammalian epithelial cells, apart from being building blocks for protein synthesis. Studies have shown that environmental factors, particularly heat stress, can negatively impact milk production performance in dairy cattle. The mammalian target of rapamycin complex 1 (mTORC1) pathway is considered the primary signaling pathway involved in regulating cell proliferation and milk protein and fat synthesis in cow mammary epithelial cells in response to amino acids and heat stress. Given the significant role played by the mTORC signaling pathway in milk synthesis and cell proliferation, this article briefly discusses the main regulatory genes, the impact of amino acids and heat stress on milk production performance, and the regulation of mTORC signaling pathway in cow mammary epithelial cells.

## 1 Introduction

The mammary gland is a highly organized organ that has the ability to transform nutrients from the bloodstream into milk constituents (Bauman et al., 2006). The quality of milk is determined by its contents, and breeders are highly concerned with the milk phenotypic traits of their animals. Several factors, such as the environment, nutrition, and endocrine factors, can affect the quantity and quality of milk produced by the mammary gland (Toerien and Cant, 2007; Hayashi et al., 2009; Burgos et al., 2010a; Burgos and Cant, 2010b; Toerien et al., 2010). For example, studies have shown that milk protein synthesis in the bovine mammary gland requires an adequate supply of amino acids and adenosine triphosphate (ATP) in the animal’s body (Pszczolkowski et al., 2020). The kinase mammalian or mechanistic target of rapamycin (mTOR) is involved in integrating various signals from the environment and translating them into specific biological responses such as milk yield and protein synthesis (Gordon et al., 2014). As a master regulator of cell development and milk synthesis, mTORC1 stimulates anabolic cellular metabolism in response to growth stimuli, nutrients, and energy (Huang and Fingar, 2014). Recent studies have also reported on the role of mTORC1 signaling in the regulation of milk contents (Ma and Blenis, 2009; Salama et al., 2019; Hu et al., 2020; Zhou J et al., 2022).

**FIGURE 1:**
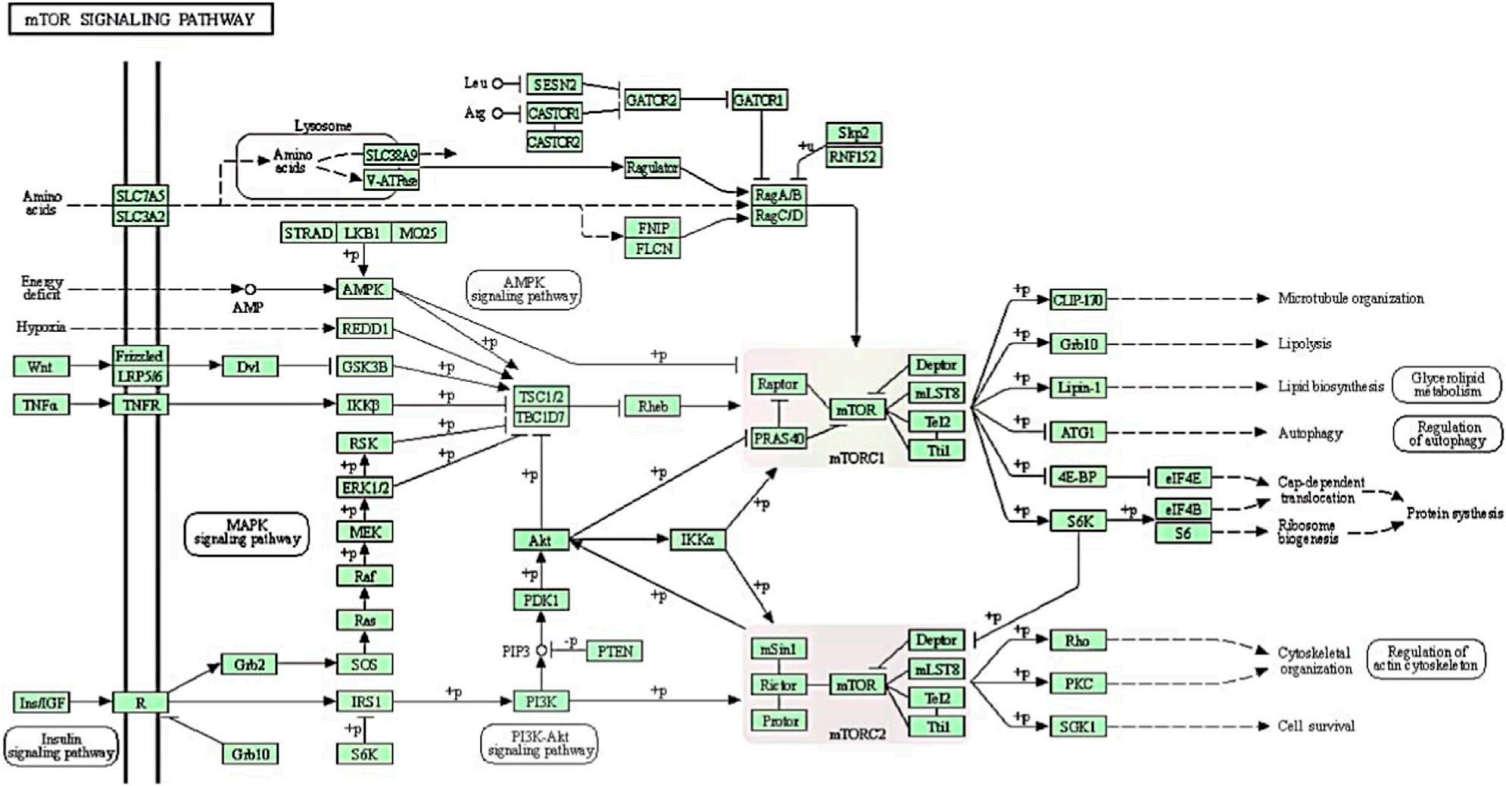
The regulatory mechanism of mTOR signaling pathways to facilitate biological functions processes such as lipid metabolism, autophagy, protein synthesis and ribosome biogenesis. mTORC1 contains mTOR, Raptor, PRAS40, Deptor, mLST8, Tel2 and Tti1. On the other hand, mTORC2, which consists of mTOR, mSin1, Rictor, Protor, Deptor, mLST8, Tel2 and Tti1, responds to growth factors and controls cytoskeletal organization, metabolism and survival. Note: +p indicates phosphorylation; −p indicates dephosphorylation; → indicates activation; 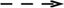
indicates indirect or unknown relationship, 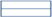
or
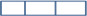
indicates complexes, 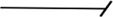
indicates repression.

The mTOR is an atypical serine/threonine kinase that is highly conserved through evolution. It plays a central role in regulating cell growth, proliferation, and metabolism in response to a variety of signals, including growth factors, nutrient availability, and stress (Sengupta et al., 2010; Jewell et al., 2013a; Jewell and Guan, 2013b). In mammalian cells, mTOR is found in two distinct protein complexes called mTORC1 and mTORC2. The activity of mTORC1 is regulated by several factors, including the intracellular energy status, external growth hormones like insulin, stress factors, and the availability of amino acids. Amino acid supplementation, environmental factors, and endocrine changes have been shown to affect the milk quantity and quality via the regulation of mTORC signaling pathway in the bovine mammary gland (Hanigan et al., 2009; Pszczolkowski et al., 2020; Zhou J et al., 2022). In this review, the underlying factors (such as genetics, environmental factors, and amino acids) that regulate the mTOR signaling pathway and how mTORC1 integrates these signals to promote milk synthesis in bovine mammary gland cells are highlighted. Figure 1 summarizes the general regulatory mechanism and biological functions performed by the mTOR signaling pathway in mammary bovine epithelial cells.

Understanding the regulation of the mTOR signaling pathway and its role in milk synthesis is critical for improving animal breeding practices and ensuring a consistent supply of high-quality milk products. This knowledge can be used to develop targeted interventions that can optimize milk production and quality in livestock.

## 2 Genetic regulation of mTOR signaling pathway for milk fat and protein synthesis

Genetic regulation of the mTOR signaling pathway in the cow mammary gland is complex and involves multiple genes and pathways. Several genes involved in regulating the mTOR pathway have been identified in cows, including the genes encoding for the mTOR protein itself, as well as its upstream regulators such as AKT and PI3K. Besides, many others genes such as sterol regulatory element-binding protein 1 (SREBP1), peroxisome proliferator-activated receptor-gamma (PPARγ), menin, stearoylcoenzyme desaturase (SCD), and fatty acid synthase (FASN) have also been documented as key regulators of mTOR signaling to facilitate its activity in milk regulation (Li, H et al., 2017a; Liu, X et al., 2021). Furthermore, it has been reported that β-sitosterol could enhance the milk protein synthesis via Janus kinase 2/signal transducer and activator of transcription 5 (JAK2/STAT5) and mTORC1 signaling (Liu, X et al., 2021). Consistently, studies have reported that mTORC1 via RPL8, FASN, SREBP1 and SCD regulates milk fat and protein synthesis (Liu, L et al., 2016; Zhou J. et al., 2021). Previous studies have found the two main signaling pathways including peroxisome proliferator-activated receptor gamma and SREBP1 are significantly associated with milk fat synthesis (Li N et al., 2014; Osorio et al., 2016; Li S et al., 2017b; Liu X. et al., 2017; He et al., 2021). The SREBP1 pathway and the PPARγ pathway are the two main pathways for milk fat synthesis.

The SREBP1 is a transcription factor that plays a crucial role in regulating lipid metabolism in the bovine mammary gland, and mTOR (mechanistic target of rapamycin) signaling has been shown to be involved in this process (Li N et al., 2014). When there is an increase in the demand for milk production in the mammary gland, SREBP1 is activated and translocates to the nucleus, where it binds to the promoter regions of genes involved in lipid metabolism and transcriptionally upregulates their expression. This results in increased *de novo* synthesis of fatty acids and triglycerides in the mammary gland, which are essential components of milk. mTOR signaling is a key pathway involved in regulating cellular metabolism and growth. It is activated by various factors, including amino acids, growth factors, and insulin, and plays an important role in regulating lipid metabolism in the mammary gland. Studies have shown that mTOR signaling is involved in the regulation of SREBP1 activity in the bovine mammary gland (Che et al., 2019). The SREBP1 responds to hormones or nutrition via mTOR signaling and then stimulates milk fat synthesis by promoting the expression of genes involved in fat synthesis, such as acetyl-CoA carboxylase alpha (ACACA), FAS and SCD1 (Li N et al., 2014). Similarly, other studies have also found that the SREBP1 plays an important role in the integrated regulation of lipid synthesis in dairy cow mammary epithelial cells through the regulation of key enzymes including fatty acid synthase, acetyl-CoA carboxylase alpha and stearoyl-CoA desaturase (Ma and Corl, 2012; Oppi-Williams et al., 2013). Studies have shown that the activation of SREBP1 is regulated by various upstream factors or pathways. Consistently, it has been reported that insulin and growth factor activate SREBP1 through phosphatidylinositol 3-kinase (PI3K)-Protein Kinase B (AKT)-mTORC1 pathway (Ricoult et al., 2016) and cholesterol activates SREBP1 through the liver X receptor (LXR) (Oppi-Williams et al., 2013; Xu et al., 2019). Specifically, mTOR signaling has been shown to activate SREBP1 by phosphorylating and inhibiting its negative regulator, insulin-induced gene (INSIG) protein. This results in the translocation of SREBP1 to the nucleus and the upregulation of genes involved in lipid metabolism. Additionally, mTOR signaling has also been shown to regulate the expression of genes involved in milk protein synthesis and secretion in the mammary gland. This is achieved through the activation of various downstream effectors, including the eukaryotic initiation factor 4E-binding protein 1 (4E-BP1) and the ribosomal protein S6 kinase (S6K1), which play important roles in regulating translation initiation and elongation. The phosphorylation of S6K and 4E-BP1 by mTOR activates the translation machinery and enhances the synthesis of proteins, including milk proteins such as casein and whey proteins, as well as enzymes involved in milk lipid synthesis. In addition, mTOR signaling also increases the expression of genes involved in milk protein synthesis and lipid metabolism by activating transcription factors such as SREBP1c.

Ribosomal protein L8 (RPL8) is another key gene involved in regulating milk fat synthesis. When RPL8 is knocked down, it reduces the expression of genes involved in lipid synthesis, resulting in decreased milk fat production. RPL8 is a component of the large ribosomal subunit and regulates milk production in the mammary gland through the mTOR signaling pathway (Lou et al., 2014). Specifically, RPL8 interacts with mTOR and enhances its kinase activity, leading to increased phosphorylation of downstream targets such as S6K and eukaryotic initiation factor 4E-binding protein 1 (4E-BP1). Previous studies have experimentally proved that RPL8 is a promising candidate gene responsible for milk fat percentage trait in dairy cattle (Jiang L et al., 2010; Jiang et al., 2015; Zheng et al., 2019). Similarly it has been evaluated through genome-wide association analysis (GWAS) that RPL8 has strong association with milk fat percentage in dairy cattle (Jiang L et al., 2010; Jiang et al., 2015). In addition, RPL8 may regulates fatty acid *de novo* synthesis and is considered promising candidate gene for the milk fat percentage trait in dairy cattle (Liu J. et al., 2017; Zheng et al., 2019).

PPARs are a class of nuclear receptors that play an important role in regulating lipid metabolism and glucose homeostasis. Bolsoni-Lopes et al. (2015) reported that PPARs and their pathways have an important regulatory role in adipocyte differentiation and adipose metabolism. Adipocyte differentiation is the process by which pre-adipocytes develop into mature adipocytes, which are the primary cells responsible for storing excess energy as fat in adipose tissue. Adipose metabolism refers to the processes involved in the uptake, storage, and release of lipids in adipose tissue. Consistently, a study has shown that cell-death-inducing DNA fragmentation factor-α-like effector A (CIDEA) by inhibiting the activity of AMP-activated protein kinase (AMPK) which is followed by enhancement of SREBP1 and PPARs which are important regulators of lipid metabolism in the mammary gland of cows, and are involved in the synthesis of milk fat (Cheng et al., 2022). Furthermore, they investigated the role of CIDEA in regulating *de novo* fatty acid synthesis in bovine mammary epithelial cells (BMECs). The authors found that CIDEA promotes *de novo* fatty acid synthesis in BMECs by regulating the AMPK/PPARγ axis and SREBP1 (Cheng et al., 2022). Specifically, CIDEA enhances the synthesis of fatty acids, which are used to produce milk fat. This effect is achieved by inhibiting the activity of AMPK, a cellular energy sensor that inhibits fatty acid synthesis, and promoting the expression of PPARγ. PPARγ is a transcription factor that activates SREBP1, another transcription factor that upregulates the expression of genes involved in fatty acid synthesis. Overall, these findings suggest that CIDEA is an important regulator of milk production in dairy cows, and that targeting the CIDEA-AMPK/PPARγ-SREBP1 pathway could be a potential strategy for improving milk production efficiency. Specifically, inhibiting AMPK activity and promoting the expression of PPARγ and SREBP1 could lead to increased fatty acid synthesis in the mammary gland, resulting in higher milk fat production. In recent studies, it has been found that lithium chloride supplementation significantly promotes milk fat synthesis in cows by regulating the activity of PPARγ, a transcription factor involved in lipid metabolism. Specifically, Zong et al. (2023) demonstrated that lithium chloride inhibits the expression of SOCS2 and SOCS3 proteins through JAK2/STAT5, mTOR, and SREBP1 signaling pathways in mammary epithelial cells, leading to increased PPARγ activity and enhanced fatty acid synthesis. These findings suggest that lithium chloride could be a potential supplement for improving milk fat production in dairy cows.

In separate studies of buffalo and dairy cattle, Liu et al. (2016) and Zhou F. et al. (2021) investigated the role of the PPARγ pathway in regulating lipid metabolism in the mammary gland. They found that the PPARγ pathway positively regulates the gene expression of fatty acid synthase, an enzyme involved in the *de novo* synthesis of fatty acids from non-lipid sources such as carbohydrates and amino acids. This suggests that the PPARγ pathway may play a positive role in regulating milk fat synthesis in cows via the mTOR signaling pathway. Overall, these studies highlight the complex signaling pathways involved in regulating milk fat synthesis in cows and suggest that targeting specific pathways, such as PPARγ and mTOR, could be a potential strategy for improving milk production efficiency. However, further research is needed to fully understand the mechanisms underlying these pathways and their effects on milk composition and quality. In addition, experimental trials is also recommended to fully understand the complex regulatory mechanisms involved in milk production in dairy cows, and to identify potential therapeutic targets for improving milk production efficiency.

ATPase family AAA-domain containing protein 3A (ATAD3A) is a protein that is encoded by nuclear genes and localizes to the mitochondrial membrane. It plays a crucial role in cellular metabolism and cell growth. ATAD3A has been implicated in the regulation of milk production in dairy cattle by modulating the mTOR signaling pathway. A recent study investigated the expression of ATAD3A in the mammary gland tissue of lactating dairy cows and found a positive correlation between its expression and milk production. The researchers also observed that ATAD3A expression was upregulated in response to mTOR signaling activation, suggesting that it may play a role in regulating milk production through this pathway (Han and Zhang, 2021). A study was conducted to investigate the impact of dietary protein on milk production in dairy cows. The results revealed that an increase in dietary protein resulted in a corresponding increase in milk production. Moreover, it was observed that the rise in milk production was linked with an increase in the expression of a protein known as ATAD3A in the mammary gland tissue. This finding supports the notion that ATAD3A plays a significant role in regulating milk production, as noted by Yang et al. (2021) in their study published in 2021. Additionally, Chen et al. (2018) conducted a study that documented the effect of extracellular amino acids and hormones on ATAD3A expression. The researchers found that these external factors significantly upregulated the expression of ATAD3A, which in turn triggered the phosphorylation of mTOR, SREBP-1c, and cyclin D1. This, in turn, led to an improvement in milk biosynthesis and cell proliferation. Overall, these findings suggest that ATAD3A may play a crucial role in regulating milk production in dairy cows, and external factors such as dietary protein and extracellular amino acids and hormones can influence ATAD3A expression and milk biosynthesis. Nuclear ubiquitous casein and cyclin-dependent kinase substrate 1 (NUCKS1) is a highly phosphorylated nuclear protein that functions as a chromatin modifier and transcriptional regulator in the mammary epithelial cells of cows. The expression of NUCKS1 is regulated by specific amino acids such as methionine and leucine, as well as hormones like estrogen and prolactin. NUCKS1 is positively associated with the activation of various signaling pathways such as the mTOR, SREBP-1c, and cyclin D1. It has been found to be critical for regulating milk protein, milk fat, and lactose synthesis, as well as cell development. A study conducted by Yuan et al. (2019) reported that NUCKS1 expression levels were positively correlated with milk synthesis and proliferation of mammary epithelial cells. Furthermore, a study conducted by Shi et al.(2020) investigated the effects of mTOR signaling inhibition on milk production in dairy cows. They found that blocking the mTOR signaling pathway led to a decrease in milk production, which was associated with a decrease in the expression of ATAD3A in mammary gland tissue. This study provided further evidence for the crucial role of NUCKS1 in regulating milk production through the mTOR pathway.

Kisspeptin-10 (KP-10) is the most effective and potent member of the Kisspeptin peptide family and its treatment increased the phosphorylation levels of mTOR and its downstream targets, including S6K1 and 4EBP1, in BMECs, indicating the activation of the mTOR pathway (Cao et al., 2021). This activation was accompanied by increased milk synthesis in BMECs. Further investigation revealed that KP-10 inhibited the expression and activity of SIRT6, a member of the sirtuin family of NAD-dependent deacetylases that can negatively regulate the mTOR pathway. The authors found that Kp-10 treatment reduced the expression of SIRT6 and increased the acetylation levels of mTOR, leading to the activation of the mTOR pathway and increased milk synthesis in BMECs. Consistently, other studies also focused on the role of KP-10 in promoting BMECs proliferation and milk synthesis through the activation of GPR54 and its downstream signaling pathways (Sun et al., 2017; Li Y et al., 2020). In the 2020 study by Li et al., the authors investigated the mechanism by which KP-10 promotes BMEC proliferation. They found that KP-10 activated GPR54 and its downstream signaling pathways, including the PI3K/Akt/mTOR pathway, leading to increased cell proliferation. Consequently, the 2017 study by Sun et al. focused on the role of KP-10 in inducing β-Casein synthesis in BMECs. The authors found that KP-10 activated GPR54 and its downstream signaling pathways, including the MAPK/ERK and PI3K/Akt/mTOR pathways, leading to increased β-Casein synthesis. Overall, these studies provide insights into the mechanisms by which KP-10 promotes BMECs proliferation and milk synthesis, and highlight the potential of KP-10 as a therapeutic target for improving milk production in dairy cows. However, further research is needed to understand the precise mechanisms involved in this process and to explore the potential side effects of using KP-10 as a milk enhancer. The summary of studies reported the genetic regulations of mTOR signaling pathway to promote milk contents in mammary gland of dairy cattle has been provided in Table 1.

**TABLE 1:** Genetic regulation of mTOR signaling pathway to promote milk contents in mammary gland of dairy cattle.

Genes	Target	Biological function	Species/tissue	Authors
IGF-1	Increased the phosphorylation of 4EBP1, mTOR and PI3K-Akt pathway	Associated with milk protein synthesis	BMECs	Bibollet-Bahena and Almazan (2009)
				Burgos and Cant (2010b)
				Wang et al. (2022c)
Kp-10	Suppressed the expression of SIRT6 and promoted the AKT, mTOR, CSN2, CSN1, GPR54 and STAT5 and enhanced the phosphorylation of mTOR signaling	Improved milk yield and casein synthesis	BMECs	Sun et al. (2017)
				Cao et al. (2021)
Mitochondrial ATAD3A	Enhanced mTORC1, SREBP-1c, and Cyclin D1	Regulates protein, fat, and lactose biosynthesis, and cell proliferation	BMECs	Chen et al. (2018)
Sirtuin 4 (SIRT4)	Upregulation of SIRT4 is positively associated with regulation of mTOR signaling pathway and also maintain the antioxidative status	Enhanced the milk production performance	BMECs	Ding et al. (2022)
PURB	Amino acids in the presence of PURB gene, stimulated mTOR signaling pathway and SREBP-1c expression	Increased milk protein and fat synthesis	BMECs	Huo et al. (2019)
Brahma (BRM)	Activated leucine-stimulated mTOR gene transcription	Promoted milk protein synthesis	MMECs	Ke et al. (2023)
14-3-3γ protein	Upregulated the expression of CSN2 and mTOR	Regulates lactogenesis in BMECs	BMECs	Khudhair et al. (2015)
Septin6	Over-expression of Septin6 enhanced the expression of CSN2, mTOR, p-mTOR, S6K1 and p-S6K	Promoted milk casein synthesis	BMECs	Li et al. (2019b)
UFL1	Increased the Phosphorylation mTOR signaling pathway followed by promoting the expression protein and fat synthesis genes (FASN, ACACA, CIDEA, CSN, WAP, SREBP 1c, Elf5 AND Cyclin D1)	Promoted milk protein and fat synthesis	BMECs	Li C et al. (2021)
Stearic acid (SAD) gene	Activates the PI3K-mTOR-4EBP1/S6K and mTOR-SREBP-1 signaling axes through the FATP4-CDK1 pathway to promote milk synthesis in BMECs	Promoted milk synthesis	BMECs	Li F et al. (2022)
CRTC2	Enhanced the m RNA expression level of mTOR signaling pathway and SREBP-1c	Milk fat synthesis	BMECs	Li et al. (2019a)
Over-expression of T1R1/T1R3	Enhanced the expression level of SLC7A5, SLC3A2 4EBP1, S6K and phosphorylation of mTOR signaling pathway	Regulated milk protein synthesis and total milk yield	MMECs	Liu et al. (2017c)
14-3-3γ protein	Enhanced the expression levels of CSN2, mTOR, S6K1, AKT1, SREBP1 and PPARγ	Promoted lactational performance	BMECs	Liu L et al. (2015)
Long non-coding RNA (lncRNA) MPFAST	Promotes BMEC proliferation and fatty acid synthesis by sponging miR-103, which in turn regulates *AKT*, *mTOR*, and *SREBP*1 followed by PI3K-AKT pathway	Promoted milk synthesis	BMECs	Liu L et al. (2022)
GRP78	GRP78 knockdown inhibited milk biosynthesis and cell proliferation and that its overexpression promoted milk biosynthesis and cell proliferation through the mTOR signaling pathway	Promoted milk synthesis	BMECs	Liu Y et al. (2019)
RagD	Regulated AA metabolism and elevated the expression level of CSN2 and mTOR signalling	Regulated milk casein synthesis	BMECs	Mu et al. (2018)
PRL and EGF	Enhanced the phosphorylation of PI3-kinase/Akt/mTOR signaling pathway	Improved the milk β-casein synthesis	MMECs	Pauloin and Chanat (2012)
Myotonic dystrophy-related Cdc42-binding kinase alpha (MRCKα)	Promoted prolactin-induced lactogenesis by activating mTOR/SREBP1/cyclinD1 signaling pathway	Enhanced milk protein and fat productivity	BMECs	Wang et al. (2022a)
METTL3	Knockdown and overexpression activated mTOR signaling pathways	Milk protein and fat synthesis	BMECs	Wang et al., 2022b
Protein 14-3-3ε	Upregulated the expression of CSN2 mTOR, p-mTOR, S6K1, and p-S6K1	Enhanced milk casein synthesis	BMECs	Wang et al. (2018b)
Cullin5 (Cul5)	Positively regulated the phosphorylation of mTOR signaling pathway	Regulated mammary cell proliferation and promoting milk yield	MMECs	Xu et al. (2022)
*NUCKS1*	mTORC1	Regulates protein, fat, and lactose biosynthesis, and cell proliferation	BMECs	Yuan et al., 2019
Amino acids and D-Glucose supplementation	Regulated JAK2/STAT5 and AMPK/mTOR signaling pathways in mammary epithelial cells of dairy cows	Milk casein synthesis	BMECs	Zhang et al. (2018a)
Zinc Finger Protein Th-POK (*ZBTB7B*)	Positively regulates Akt-mTOR-SREBP signaling pathway	Improved lactogenesis in mammary gland	MMECs	Zhang et al. (2018b)

## 3 Factors causing reduction of milk production performance via inhibition of mTOR signaling pathway in dairy cattle

### 3.1 Genetic factors role in the inhibition of mTOR signaling pathway and reduction of milk production performance in dairy cattle

The genetic factors involved in inhibiting the mTOR signaling pathway and reducing milk production have been summarized in Table 2. It has been found that over-expression of certain genes can suppress milk yield, milk fat, and protein synthesis by blocking the activation of the mTOR signaling pathway. Most of these genes are involved in inhibiting the stimulation of amino acids on mTOR gene transcription (Huang and Fingar, 2014; Lin G et al., 2023). For instance, ARID4B, a member of the DNA binding AT-rich interactive domain (ARID) family, has been shown to attach to the promoter region of mTOR and block the attachment of methionine, which can result in the suppression of milk protein synthesis (Lin G et al., 2023). Another study reported that ARID1A inhibits milk protein synthesis in the mammary gland by impairing the signaling from amino acids (Met and Leu) to the mTOR pathway (Qi et al., 2022).

**TABLE 2:** Summary of factors (genetic, nutritional and environmental) causing inhibition of mTORC signaling pathways and consequent low milk production performance in dairy cattle.

Agent	Target	Biological function	Species/tissue	Authors
*S. aureus*	Suppressed the mTOR signaling pathway followed by downregulated expression of SLC1A3 and SLC7A5	Reduced the milk yield and milk protein synthesis	BMECs	Chen Y et al. (2021)
Deficiency of AA	Causes deactivation of mTOR signaling pathway	Impaired milk protein yield	BMECs	Doelman et al. (2015a)
*MEN1*/Menin	Over-expression of MEN1 downregulated the level of CSNK, PRL and mTOR signaling pathway	Decreased milk protein synthesis	BMECs	Li H et al. (2017a)
SECN2	Over-expression of SECN2 inhibited the expression level of mTOR signaling	Decreased milk casein synthesis	BMECs	Luo et al. (2018a)
Sestrins (SECN2)	Suppressed the expression of mTORC1 and AA-mediated β-casein (CSN2) synthesis via SH3BP4 and ATF4	Inhibited milk casein synthesis	BMECs	Luo et al. (2019a)
ARID1A	The over-expression of ARID1A gene blocked the action of Met and Leu on mTOR signaling pathway	Suppressed milk synthesis	BMECs	Qi et al. (2022)
High level of fatty acids in ketosis	Regulated JAK2/STAT5 and AMPK/mTOR signaling pathways in mammary epithelial cells of dairy cows	Suppressed milk casein synthesis	BMECs	Shu et al. (2020)
Pten	Over expression of Pten gene significantly downregulated AKT, MTOR, S6K1, STAT5, SREBP1, PPARγ, PRLR, and GLUT1	Reduced lactational performance	BMECs	Wang et al. (2014c)
Melatonin	Melatonin suppressed the expression mTOR signaling pathway via MT1 receptor	Suppressed milk yield and milk fat synthesis	BMECs	Wang et al. (2019b)
Bta-miR-34b	Restricted the phosphorylation of Akt/mTOR signaling pathway signaling	Reduced milk fat biosynthesis	BMECs	Wang Y et al. (2021)
D-glucose and amino acids	Regulated JAK2/STAT5 and AMPK/mTOR signaling pathways in mammary epithelial cells of dairy cows	Suppressed milk casein synthesis	BMECs	Zhang et al. (2018a)

*MEN1* is a key gene encoding protein menin that was documented to have a role in milk protein synthesis in mammary epithelial cells (Li H et al., 2017a). Furthermore, they documented that the over expression of menin was negatively correlated with κ-casein (CSNK), prolactin (PRL) and/or insulin (INS) and followed by downregulation of mTOR signaling resulting in lower milk protein synthesis in mammary epithelial cells (Li H et al., 2017a). Similarly, another study found that melatonin significantly compromised the milk fat synthesis by inhibiting the regulation of mTOR signaling in mammary epithelial cells (Wang Y. et al., 2019). Overall, the findings revealed that menin protein interact with mTOR and its downstream signaling components in mammary epithelial cells. This interaction leads to the inhibition of mTOR signaling pathway, which is a crucial regulator of milk protein synthesis in these cells. The overexpression of menin protein in mammary epithelial cells has been associated with a decrease in the expression of κ-casein, prolactin, and insulin, which are essential factors for milk protein synthesis. This downregulation of these factors leads to a decrease in the activation of mTOR signaling, leading to a reduction in milk protein synthesis.

Melatonin has been shown to inhibit mTOR signaling in mammary epithelial cells, leading to a reduction in milk protein and fat synthesis (Wang Y. et al., 2019). This suggests that melatonin may have an inhibitory effect on milk production in dairy cows. Furthermore, the inhibitory effect of melatonin on milk fat synthesis was found to be mediated by the MT1 receptor, which is a receptor for melatonin (Wang Y. et al., 2019). One possible explanation for this effect is that melatonin regulates the expression of genes involved in milk synthesis and secretion by modulating mTOR signaling. Another possibility is that melatonin affects the secretion of other hormones, such as prolactin, which is an essential regulator of milk production in dairy cows. In addition, melatonin may reduce milk production in dairy cows by inhibiting mTOR signaling in mammary epithelial cells, which leads to a decrease in milk protein and fat synthesis. Further research is needed to fully understand the mechanisms underlying the effects of melatonin on milk production in dairy cows.

MicroRNAs (miRNAs) are small non-coding RNAs that regulate gene expression post-transcriptionally by binding to the 3′untranslated region (UTR) of target mRNAs. miRNAs have been shown to play important roles in regulating milk synthesis in mammary epithelial cells (Wang Y et al., 2021). One mechanism by which miRNAs can reduce milk production is through the mTOR signaling pathway. Specifically, miRNAs can inhibit the expression of key genes (PPARγ, FASN, Akt, mTOR, IGF2, Raptor, S6K1 and TNS1) which are involved in the activation of mTOR pathway. This inhibition can lead to a decrease in milk protein synthesis and a reduction in milk production (Wang Y et al., 2021). Consequently, a study by Wang M et al. (2021) investigated the effect of lipopolysaccharide (LPS) on triglyceride synthesis in dairy cow mammary epithelial cells. They found that LPS can inhibit triglyceride synthesis by upregulating microRNA-27a-3p (miR-27a-3p), which targets the peroxisome proliferator-activated receptor gamma (PPARG) gene. PPARG is a transcription factor that plays a critical role in regulating milk fat synthesis in mammary epithelial cells. Recent studies have suggested that the inhibitory effect of lipopolysaccharide (LPS) on triglyceride synthesis may be mediated by miR-27a-3p, which targets and downregulates PPARG expression (Wang M et al., 2021). This suggests that miR-27a-3p may play a crucial role in regulating milk fat synthesis under conditions of inflammation or stress. Similarly, microRNA-432 has also been identified as a regulator of milk fat synthesis in ovine mammary epithelial cells (Hao et al., 2021). The authors found that microRNA-432 inhibits milk fat synthesis by targeting SCD and lipoprotein lipase (LPL), which are essential for fatty acid synthesis and uptake, respectively. These findings suggest that microRNA-432 could be a potential target for improving milk composition in dairy animals.

Interestingly, it has been noticed that high fatty acids supplementation in dairy cattle during periparturient period also compromised the milk casein synthesis by suppressing the mTOR activity in mammary gland (Shu et al., 2020). Furthermore, they documented that high fatty acids attenuated the positive impact of methionine and prolactin on mTOR signaling which result in reduction of milk casein synthesis (Shu et al., 2020). Consistently Zhang et al. (2018a) also observed that deficiency of d-glucose and amino acids were associated with declining activity of mTOR, stat5a, 4EBP1 and S6K1 in mammary gland. Furthermore, they found that the synthesis of milk protein was significantly compromised in bovine mammary epithelial cells.

### 3.2 Heat stress compromise dairy cattle milk production performance via reduced phosphorylation of mTOR signaling

Hyperthermia or heat stress is a common stressor that dairy animals may experience during hot summer months, which can negatively impact their production performance. Recent studies have investigated the effects of heat stress on the mTOR signaling pathway and its link with milk synthesis in dairy animals. Heat stress has been shown to decrease the expression levels of mTOR signaling pathway-linked genes, such as ribosomal protein S6 (RPS6), AKT serine/threonine kinase 1 (AKT1), and ribosomal protein S6 kinase B1 (RPS6KB1), in bovine mammary epithelial cells (Zhou J et al., 2022). The downregulation of these genes leads to the suppression of mTOR signaling, which can have a negative impact on milk synthesis in the mammary gland of dairy animals. Furthermore, in goat mammary glands, the phosphorylation of several proteins, including mTORC1, was found to be upregulated in response to lactation-related signaling pathways such as calcium signaling and oxytocin signaling pathways (Zhu C et al., 2021). These findings suggest that these signaling pathways play a key role in the regulation of lactation in dairy animals. Fu L et al. (2021) discovered that heat stress can have a negative impact on milk production in bovine mammary epithelial cells (BMECs) by suppressing the expression of genes associated with the mTORC1 signaling pathway, including Ras homolog enriched in the brain (Rheb), AKT, eIF4E, and eEF2K. This finding was further supported by Guo Z et al. (2021). In addition; Ding Q et al. (2022) reported a significant decrease in the expression of the SIRT4 gene in response to heat stress, which was associated with increased oxidative stress and decreased phosphorylation levels of the mTORC1 signaling pathway. As a result, the reduced expression of SIRT4 gene due to oxidative stress was found to be the main factor responsible for decreased milk protein synthesis and milk yield in BMECs. In conclusion, these studies suggest that heat stress can downregulate the expression of genes associated with the mTORC1 signaling pathway, leading to poor milk production performance in BMECs (Table 2).

In summary, we concluded that not only genetic factors but both environmental and nutritional management practices can also affect mTOR signaling and enhance milk or fat synthesis. Here are some practices that can support milk or fat synthesis through mTOR signaling pathway.

Feeding high-quality protein is an essential nutrient required for milk synthesis, and feeding high-quality protein sources can stimulate mTOR signaling and enhance milk production. The amino acid leucine, in particular, is known to activate mTOR signaling and promote milk synthesis. Providing adequate energy: Adequate energy intake is crucial for milk synthesis, and underfeeding can suppress mTOR signaling and reduce milk production. Ensuring that lactating animals receive enough energy through their diet can support milk or fat synthesis.

Optimizing feeding frequency and timing (i.e., 3–4 times/day) can increase mTOR signaling and enhance milk synthesis compared to infrequent feeding (i.e., 1–2 times/day). Additionally, feeding before or after milking can affect milk production with some evidence, suggesting that feeding after milking can improve milk yield.

Providing a comfortable environment is needed because environmental stressors, such as heat or cold stress, can suppress mTOR signaling and reduce milk production. Providing a comfortable environment that minimizes stress can help support milk or fat synthesis. It is important to note that the specific management practices that enhance milk or fat synthesis through the mTOR signaling pathway may vary depending on the animal species, breed, and individual characteristics.

## 4 Amino acids supplementation regulates milk production via mTOR signaling pathway

The positive role of nutrition in improvement of milk production performance in ruminants has been extensively discussed in a recent study (Abdelrahman et al., 2022). Consistently it is well-established that amino acids are taken up from the bloodstream into the mammary gland cells by specific transporters. Amino acids act as a signal to activate the mTOR pathway by binding to intracellular sensors, such as the Rag GTPases and the leucine sensor Sestrin2. The activated mTOR pathway leads to the formation of the mTORC1, which is a key regulator of protein synthesis and cell growth. Furthermore, mTORC1 activation leads to the phosphorylation of downstream targets, such as the S6K and 4EBP, which in turn activate milk synthesis in mammary glands of dairy cattle.

Several studies have identified amino acids as important supplements for regulating cell proliferation, protein synthesis, and other physiological processes through the mTORC1 signaling pathway (Sancak et al., 2008; Sengupta et al., 2010; Lei et al., 2012; Wang F. et al., 2019). In the presence of sufficient amino acid supply, mTORC1 is activated and translocates to the lysosomal surface, where it triggers the phosphorylation of mTOR (Sancak et al., 2008). *In vitro* studies have shown that the supplementation of essential amino acids (EAA) can effectively regulate milk protein synthesis in BMECs through the activation of the mTOR signaling pathway (Toerien et al., 2010; Appuhamy et al., 2011; Appuhamy et al. 2012; Appuhamy et al. 2014). These studies demonstrated that EAA supplementation can increase the phosphorylation of mTOR, S6K1, eIF4E, 4EBP1, and insulin receptor substrate 1 (IRS1), leading to enhanced milk protein synthesis in BMECs. Furthermore, insulin treatment has also been found to enhance the phosphorylation of mTOR, Akt, S6K1, 4EBP1, and IRS1, indicating the importance of insulin signaling in regulating milk protein synthesis (Appuhamy et al., 2011; Appuhamy et al. 2012; Appuhamy et al. 2014).

The mammary gland utilizes amino acids as substrates for milk synthesis through various signaling pathways, including the mTORC1 (Appuhamy et al., 2014). Amino acids such as leucine, isoleucine, methionine, and threonine have been shown to function as signaling molecules that favorably control milk synthesis and lactation in earlier investigations on cows and mice (Trottier, 2014). As upstream activators of mTORC1, amino acid sensors, such as vacuolar H+-ATPase, SLC38A9, Sestrin2, CASTOR1 homodimer, and CASTOR1-CASTOR2, are thought to regulate milk production and lactation (Stransky and Forgac, 2015; Chantranupong et al., 2016; Gai et al., 2016). The mTOR pathway regulates its targets after activation to facilitate milk protein synthesis (Gao et al., 2015). Previous studies have demonstrated that high temperature (42°C) incubation of bovine mammary cells inhibited protein translation by decreasing the activity of the mTOR downstream pathway (Kaufman et al., 2018; Salama et al., 2019). Additionally, Nan et al. (2014) experimentally demonstrated that a 2.9:1 ratio of Lys: Met supply positively regulates the *in vitro* synthesis of casein by targeting mTORC1 signaling in bovine mammary epithelial cells. Similarly, another study found that methionine and arginine supply could significantly contribute to milk protein synthesis in bovine mammary epithelial cells via the mTORC1 pathway (Hu et al., 2020). They documented that an increased supply of methionine and arginine upregulated mTORC1 signaling by targeting the SLC7A1 gene. Consistently, *in vitro* findings have shown that amino acid supplementation enhances the phosphorylation of the mTORC1 signaling pathway, which further regulates the synthesis of milk protein in mammary gland cells (Appuhamy et al., 2011). In addition, the mammary epithelial cells were treated with a mixture of essential amino acids, and the rate of milk protein synthesis was measured. The researchers observed that amino acid treatment enhanced the phosphorylation of mTORC1 and downstream targets, such as S6K1 and 4EBP1, leading to increased milk protein synthesis (Appuhamy et al., 2011). In summary, these studies suggest that amino acids, including leucine, isoleucine, methionine, and threonine, play important roles in regulating milk protein synthesis via the mTORC1 signaling pathway in mammary epithelial cells. Additionally, amino acid sensors and gene regulators such as SLC7A1 play key roles in controlling milk production and lactation.

Studies have shown that amino acid treatment can enhance mammary protein synthesis rate and phosphorylation of mTORC1 in mammary epithelial cells. In particular, methionine and valine have been found to activate mTORC1 via the heterodimeric amino acid taste receptor TAS1R1/TAS1R3 and intracellular Ca^2+^ in bovine mammary epithelial cells, further facilitating milk synthesis (Zhou Y et al., 2018). In a study conducted by Zhou Y et al. (2018), it was found that knockdown of TAS1R1 and TAS1R3 significantly reduced the phosphorylation of mTORC1 and lowered the biosynthesis of β-casein in bovine mammary epithelial cells. This indicates that TAS1R1/TAS1R3 regulates the extracellular amino acids and mTORC1 pathway, resulting in elevated levels of Ca^2+^ concentration, β-casein, and protein in bovine mammary epithelial cells. These findings suggest that the TAS1R1/TAS1R3 receptor plays a critical role in regulating the mTORC1 pathway and milk synthesis in dairy cows. However, further research is needed to fully understand the complex interplay between amino acid receptors, the mTOR pathway, and other regulatory factors that may influence milk synthesis in mammary epithelial cells. A deficiency of amino acids and D-glucose has been found to significantly reduce the phosphorylation of mTORC1, AMP-activated protein kinase (AMPK), and the Janus kinase (Jak)-signal transducer and activator of transcription (Stat) signaling pathway in bovine mammary epithelial cells, as demonstrated in a study by Zhang et al. (2018a). For ease, the summary of studies evaluated the effect of amino acids supplementation on milk production performance in dairy cattle mammary epithelial cells by regulation mTORC signaling pathway has been highlighted in Table 3.

**TABLE 3:** Summary of studies on amino acids supplementation effect on bovine milk production performance via regulation of mTORC signaling pathway in dairy cattle.

Agent	mTORC1 signaling pathway as a target	Biological function	Species/tissue	Authors
Isoleucine, leucine, methionine, and threonine	Enhanced the phosphorylation of mTOR EEF1A1, S6K1,EEF2, RPS6KB1, EIF4EBP1 and 4EBP1	Improve milk and milk protein	BMECs	Apelo et al. (2014)
Amino acid supplementation	Increased the phosphorylation of mTOR, Akt, S6K1, IRS1, and 4EBP1	Improved the milk protein synthesis	BMECs	Burgos et al. (2010a)
				Appuhamy et al. (2011)
				Appuhamy et al. (2012)
				Appuhamy et al. (2014)
Amino acid supplementation	Enhanced the expression level of phosphorylated Akt, mTOR and AMPK gene	Promoted the milk casein synthesis rates	BMECs	Castro et al. (2016)
SARS	Elevated the mRNA level of GCN2, CCND1 and mTOR signaling pathway phosphorylation	Increased cell proliferation, the total milk protein synthesis and β-casein production	BMECs	Dai et al. (2018)
				Dai et al. (2020)
Amino acid supplementation	Upregulated the expression of S6K1 and eIF2Bε and phosphorylation of mTOR signaling pathway	Enhanced milk protein yield	BMECs	Doelman et al. (2015b)
Amino acids supplementation	Enhanced the expression level of Akt*, SLC7A5*, *SLC36A1*, *SLC38A2*, *SLC38A9*,*SLC43A1, CSN2* and phosphorylation of mTOR signaling pathway	Promoted milk protein synthesis	BMECs	Dong et al. (2018b)
Amino acids feeding	Enhanced the mRNA expression of β-casein and AA transporters (SLC7A5, SLC36A1, SLC38A2, SLC38A9, and SLC43A1) and elevated the mTOR phosphorylation	Enhanced milk protein	BMECs	Dong et al. (2018a)
Leucine and histidine	Upregulated the expression of mTOR, Raptor, S6K1, 4EBP1, RPS6, eIF4E and eEF2 followed by increasing the phosphorylation of mTOR signaling pathway	Enhanced the milk protein synthesis	BMECs	Gao et al. (2015)
His, Lys, Met, and Leu treatment	Elevated the expression of β-casein, S6K1 and 4EBP1 and enhanced the phosphorylation of mTOR signaling pathway	Improved milk casein and milk protein synthesis	BMECs	Gao et al. (2017)
Arg and Met supply	Enhanced the activity of mTORC1 signaling by targeting SLC7A1	Increased milk protein synthesis	BMECs	Hu et al. (2020)
Leucine and methionine supplementation	Enhanced the expression of PURB which further stimulate mTOR signaling pathway and SREBP-1c activity	Promoted milk protein and fat synthesis	BMECs	Huo et al. (2019)
Amino acid supplementation	Upregulated the phosphorylation level of mTOR signaling pathway	Milk and Milk Protein Production	BMECs	Kim and Lee (2021)
Amino acid supplementation	Enhanced the mRNA expression of SLC1A5, SLC7A5, Akt, S6K1, IRS1, EEF1A1, EEF2, EIF4EBP1, *RPS6KB1* and phosphorylation of mTOR	Improved the milk protein synthesis	BMECs	Li et al. (2017b)
Leucine and methionine supplementation	Upregulated the level of CRTC2 followed by elevated the expression of mTOR signaling pathway and SREBP-1c	Increased milk fat synthesis	BMECs	Li et al. (2019a)
Lysine supplementation	Regulated the phosphorylation level of mTOR and JAK2-STAT5 signaling pathways	Enhanced milk protein synthesis	BMECs	Lin et al. (2018)
Methionine, Liucine, isoleucine or threonine supplementation	Upregulated the expression of 4EBP1, Akt and promoted the phosphorylation of mTOR signaling pathway	Enhanced milk protein synthesis	MMECs	Liu et al. (2017a)
Amino acid supplementation	Suppressed the expression level of SECN2 and enhanced the phosphorylation of mTOR signaling	Enhanced milk casein synthesis	BMECs	Luo et al. (2018a)
Leucine treatment	Upregulated the expression of Guanine nucleotide-binding protein subunit gamma-12 (GNG12) via activation of mTOR signaling pathway	Enhanced milk β-casein synthesis	BMECs	Luo C et al. (2018b)
glycyl-tRNA synthetase (GlyRS)	GlyRS expression and activity were increased in response to amino acid stimulation in bovine mammary epithelial cells	Improved milk synthesis	BMECs	Luo et al. (2019b)
	Knockdown of GlyRS expression decreased amino acid-induced milk synthesis in bovine mammary epithelial cells, while overexpression of GlyRS increased milk synthesis			
	GlyRS-mediated milk synthesis is regulated by the mTOR signaling pathway			
Amino acid supplementation	Inhibited the SECN2, SH3BP4 and ATF4 expression and upregulated the level of mTORC1 and AA-mediated β-casein (CSN2) synthesis	Promoted milk casein synthesis	BMECs	Luo et al. (2019b)
Methionine treatment	Regulated phosphorylation of mTORC signaling pathway and increased the expression level of SLC7A1, SLC38A1, SLC38A2, ATF6 and PPP1R15A	Enhanced milk yield and milk protein synthesis	BMECs	Ma et al. (2019)
Lysine and methionine supplementation	The expression of genes (CSN1S1, CSN1S2, CSN2, CSN3, LALBA, JAK2, STAT5, ELF5 and mTOR) and phosphorylation of pathways (JAK2-STAT5 and mTOR) were enhanced	Enhanced milk yield and milk protein synthesis	BMECs	Nan et al. (2014)
Amino acids restriction	Reduced mTORC1 signaling activity	Decreased milk production performance in mice mammary gland	BMECs	Pszczolkowski et al. (2020)
Leucine treatment	Enhanced the phosphorylation level of mTOR signaling pathway and SREBP-1c by up-regulating the level of DDX59	Promoted synthesis of milk	BMECs	Qiu et al. (2019)
Supplementation of methionine and arginine	Reversed the suppression of MTOR,AKT, EIF4EBP1, *MAPK1*, *SREBF1*, *RPS6KB1*, *JAK2, SLC1A5* and *SLC7A1* caused by heat stress in cows mammary gland	Enhanced milk production performance	BMECs	Salama et al. (2019)
lysine/methionine and glucose levels supplementation	Elevated the expression of ELF5, CSN1S1, *JAK2*, and *RPS6KB1* and phosphorylation of mTOR signaling pathway were significantly increased	Improved milk casein synthesis	BMECs	Wang et al. (2019a)
Leucine treatment	Increased the mRNA expression of S6K1, GLUT1, Cyclin D1, SREBP-1c and also elevated the phosphorylation of mTOR signaling pathway	Regulated the lactogenic performance of dairy cattle	BMECs	Wang L et al. (2014b)
Arginine treatment (369.65 mg/L-729.64 mg/L in culture medium)	Enhanced the expression level of JAK2, STAT5 and casein genes (CSN1S2, CSN3, S6K) and elevated the phosphorylation of mTOR signaling	Enhanced the milk casein synthesis	BMECs	Wang M et al. (2014a)
Valine supplementation	Promoted the activity of BCAT2 followed by activation of mTOR signaling pathway	Enhanced milk β-casein synthesis	BMECs	Wang et al. (2022a)
AA supplementation	Enhanced the expression of T1R1 and T1R3 following elevated phosphorylation of mTOR, S6K, and 4EBP1	Improved milk protein synthesis	MMECs	Wang Y et al. (2017)
Methionyl-Methionine treatment	Promoted the phosphorylation of JAK2-STAT5 and mTOR signaling pathways	Enhanced milk protein synthesis	BMECs	Yang J et al. (2015)
				Wang et al. (2018a)
Amino acids supplementation	Regulated the mRNA expression of 4EBP1 and enhanced the phosphorylation of mTOR signaling pathway	Enhanced milk protein synthesis	BMECs	Yoder et al. (2019)
Amino acids and D-Glucose supplementation	Regulates JAK2/STAT5 and AMPK/mTOR signaling pathways in mammary epithelial cells of dairy cows	Enhanced milk casein synthesis	BMECs	Zhang et al. (2018a)
Supplementation of methionine in combination with EAA	Increased the phosphorylation level of eEF2, 4eBP1 and mTOR signaling pathway	Increased milk protein yield	BMECs	Zhao et al. (2019)
Amino acids supplementation	Enhanced the expression level of 4EBP1, eEF2 and upregulated phosphorylation level of mTOR signaling pathway	Promoted milk protein synthesis	BMECs	Zhao K et al. (2019)
Methionine supplementation	Positively regulated the AKT-mTOR-RPS6KB1 signalling pathway signaling pathway and its related genes (RPS6KB1, CSN1S1, CCN2, SLC38A2, SLC38A3, AKT)	Promote the synthesis of milk proteins	BMECs	Zhou et al. (2021a)
Methionine and valine	Enhanced the phosphorylation of mTOR, and elevated the expression of 4EBP1, S6K1, TAS1R1, TAS1R3	Improve milk production performance, milk calcium level and casein	BMECs	Zhou Y et al. (2018)

## 5 Conclusion

The mTOR signaling pathway is a crucial regulatory pathway in cells that plays an essential role in controlling various cellular processes such as protein synthesis, cell growth, and proliferation. It is a conserved pathway that is activated by multiple signals such as growth factors, amino acids, energy status, and stress. The process of milk biosynthesis in cow mammary epithelial cells is dependent upon a consistent supply of energy and nutrients, specifically in the form of amino acids. As the fundamental building blocks of proteins, amino acids play a critical role in the regulation of the mTOR pathway. Through the activation of key upstream regulators including mTOR, S6K1, eIF4E, 4EBP1, FASN, Akt, IGF2, Raptor, Sestrin2, CASTOR1, CASTOR2, S6K1, TNS1, and Rheb, amino acids stimulate the mTORC1 complex, ultimately driving milk biosynthesis. Environmental factors such as heat stress inhibit the activity of mTORC1 by reducing the levels of amino acids resulting in poor milk production performance. Understanding the mechanism of mTOR regulation by amino acids and heat stress can help to develop strategies to enhance milk production in cows under heat stress conditions.

## Abbreviations

CCND1, Seryl-tRNA synthetase (SARS), cyclin D1; 4EBP1, eukaryotic initiation factor 4E binding protein 1; eEF2, elongation factor 2; TAS1R1/TAS1R3, AA taste 1 receptor member 1/3; mTOR, mechanistic target of rapamycin; AKT, eukaryotic initiation factor 2a, serine-threonine protein kinase; EIF4EBP1, 4E binding protein 1; MMECs, Mouse mammary epithelial cells; BCAT2, branched-chain aminotransferase transaminase 2; EAA, Essential amino acid; ATAD3A, ATPase family AAA-domain containing protein 3A; NUCKS1, Nuclear ubiquitous casein and cyclin-dependent kinase substrate 1; BMECs, bovine mammary epithelial cells; (IGF)-1, insulin-like growth factor; PRL, Kisspeptin-10 (Kp-10), Prolactin; EGF, Epidermal growth factor; MMECs, mouse mammary epithelial cells; GLUT1, Zinc Finger and BTB Domain Containing 7B, glucose transporter 1; UFL1, Ufmylation; PURB, purine-rich element binding protein B; CRTC2, cAMP response element-binding protein-regulated transcription coactivator 2; METTL3, Methyltransferase-like 3; GRP78, Glucose-Regulated Protein 78; CSN2, AA-mediated β-casein; SESN2, Sestrin2; SH3BP4, SH3 domain-binding protein 4; ATF4, Activating transcription factor 4; CSNK, milk protein κ-casein; ARID1A, AT-rich interaction domain 1A.
